# Three new species of *Atopsyche* Banks (Trichoptera, Hydrobiosidae) from Brazil

**DOI:** 10.3897/zookeys.207.3419

**Published:** 2012-07-11

**Authors:** Allan P. M. Santos, Ralph W. Holzenthal

**Affiliations:** 1 Departamento de Zoologia, Instituto de Biologia, Universidade Federal do Rio de Janeiro, P.O. box 68044, Rio de Janeiro, RJ 21941-971, Brazil; 2 CAPES Foundation, Ministry of Education of Brazil, Brasília, DF 70040-020, Brazil; 3 Department of Entomology, University of Minnesota, 219 Hodson Hall, 1980 Folwell Ave., St. Paul, Minnesota, 55108, USA

**Keywords:** Atlantic forest, *Atopsyche*, Brazil, caddisfly, Hydrobiosidae, Neotropics

## Abstract

Three new species of *Atopsyche* Banks (Hydrobiosidae) from Brazil are described and illustrated: *Atopscyhe (Atopsaura) blahniki* **sp. n.**, *Atopsyche (Atopsyche) parauna* **sp. n.**, and *Atopsyche (Atopsaura) galharada* **sp. n.** Additional illustrations of the male genitalia of *Atopsyche urumarca* Schmid are provided, including its populational variation. Also, we provide new state records for 2 species: *Atopsyche (Atopsyche) urumarca* from São Paulo, and *Atopsyche (Atopsaura) plancki* Marlier from Santa Catarina.

## Introduction

*Atopsyche* Banks constitutes the most diverse genus in the family Hydrobiosidae, with over 120 described species (Holzenthal and Cressa 2002, Holzenthal et al. 2007). The genus occurs from the southwestern United States to northern Argentina, but it is replaced by other genera of Hydrobiosidae in the Chilean subregion (Holzenthal and Cressa 2002). Schimd (1989) provided a world revision of the family Hydrobiosidae, including comments on the classification and phylogeny of *Atopsyche* and descriptions of several new species. Currently, 19 species are recorded from Brazil, of which only 2 were recorded after Schmid (1989): *Atopsyche chirihuana* Schmid, originally described from Ecuador; and *Atopsyche erigia* Ross, originally described from Mexico (Blahnik et al. 2004). Following Schmid (1989), *Atopsyche* species are divided into three subgenera, based on features of the male genitalia: *Atopsaura* Ross, *Atopsyche* Banks, and *Dolochorema* Banks.

In this paper, we describe 3 new species of *Atopsyche* from southeastern Brazil. These additional species bring the number of known caddisflies species from Brazil to 569, but many species of this and other genera remain undescribed. In addition, we illustrate variations in the male genitalia of *Atopsyche urumarca* Schmid, and provide new state distributional records of *Atopsyche urumarca* from São Paulo and *Atopsyche plancki* Marlier from Santa Catarina.

## Material and methods

Morphological terminology used in this paper follows that presented by Schmid (1989). The lactic acid method (Blahnik et al. 2007) was used for specimen preparation. Genital structures were observed and illustrated with a compound microscope, equipped with a drawing tube. Pencil sketches were scanned and placed into an Adobe Illustrator (v. 13.0.0, Adobe Systems Inc.) document to produce a digital illustration. Descriptions provided for new species were made using the software DELTA (Dallwitz et al. 1999). Holotypes are deposited in the Museu de Zoologia, Universidade de São Paulo, São Paulo, Brazil (MZSP). Paratypes and other material examined are deposited in the MZSP and also in the University of Minnesota Insect Collection, St. Paul, Minnesota, USA (UMSP), the National Museum of Natural History, Smithsonian Institution, Washington DC, USA (NMNH), and the Coleção Entomológica Prof. José Alfredo Pinheiro Dutra, Universidade Federal do Rio de Janeiro, Rio de Janeiro, Brazil (DZRJ).

## Taxonomy

### Atopsyche (Atopsaura) blahniki sp. n.

urn:lsid:zoobank.org:act:17398E47-24AC-45A6-8FEF-CF2B3A9BBFE2

http://species-id.net/wiki/Atopsyche_blahniki

Figs 1 , 4 , 7

#### Diagnosis.

This new species is most similar to *Atopsyche zernyi* Flint, also described from Brazil. Both species have an apical process on the first article of the inferior appendage as long as the second article, and a phallotheca with long paired processes, apically upturned and narrow. However, *Atopsyche blahniki* sp. n. differs from *Atopsyche zernyi* by the narrower parapod, longer filipod (exceeding length of parapod), and in the apices of the paired processes from the phallotheca, which bear several spines on their lateral edges.

#### Male.

Forewing length 5.5 mm (n=1). Overall body color brown; antennal scape brown, with long brown setae, pedicel brown, basal flagellomeres yellow, apical flagellomeres brown; setae of palps yellow; frons and vertex of head with long, erect brown and whitish setae; legs yellowish brown, coxae and femora of forelegs darker brown. Forewings brown; erect setae on veins forming irregular pattern of alternate dark brown and yellow setae; apex of wing with fringe of alternating patches of dark brown and yellow setae. Forewing venation complete (Fig. 4A); R1 branched; stem of fork I about twice its length; fork II long, sessile; stem of fork III equal to its length; fork IV long, sessile; stem of M almost straight between m-cu crossvein and first fork of M; fork V long, narrow; Cu2 long, converging near fused anal veins; apex of fused anal veins very short. Hind wing (Fig. 4B) with Sc and R1 fused apically; forks I, III, and V present, the first with a short stem, the last long with a short stem, stem of fork III equal to its length; forks II and IV absent; M3+4 not reaching wing margin; Cu2 long and almost straight; 1A long and sinuate. Nygmas indistinct in both wings. Tergum II with pair of prominent glands at posterolateral margin (Fig. 7A); tergum III with pair of prominent gland at anterolateral margin, lined at opening with minute setae (Figs 7A, 7B). Sternum V with pair of small, rounded glands on anterolateral margins (Fig. 7C). Sterna VI and VII with prominent spine-like ventral processes on posteromesal margins.

#### Male genitalia.

**Figure 1.**
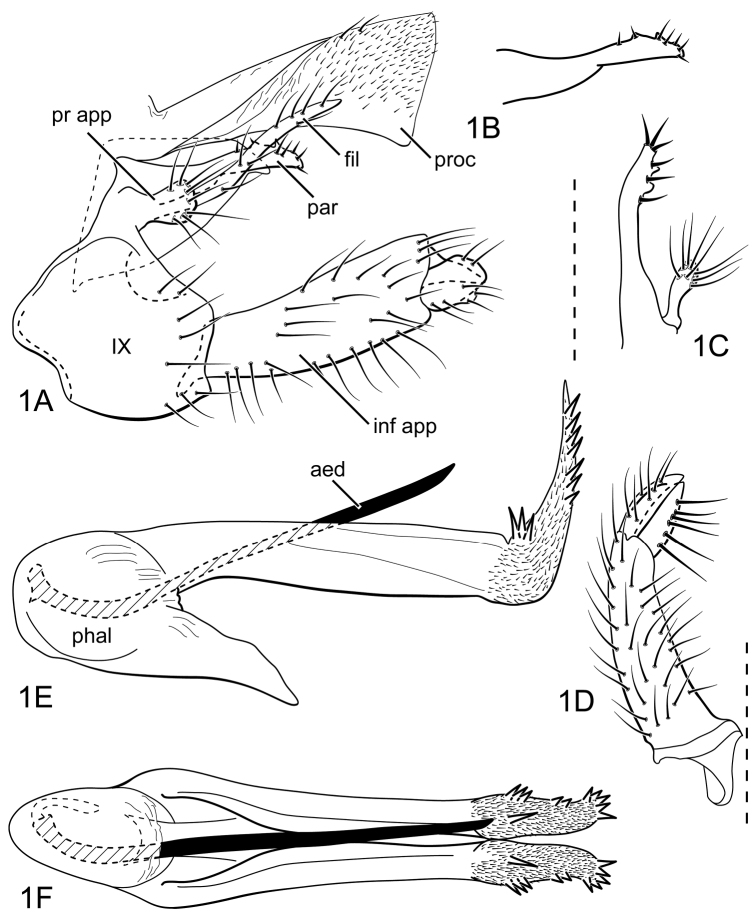
*Atopsyche (Atopsaura) blahniki* sp. n. Male genitalia: **A** lateral **B** parapod, lateral **C** parapod and preanal appendage, dorsal **D** inferior appendage, ventral **E** phallic apparatus, lateral **F** phallic apparatus, dorsal. Abbreviations: **aed** aedeagus **fil** filipod **inf app** inferior appendage **par** parapod **phal** phallotheca **pr app** preanal appendage **proc** proctiger **IX** abdominal segment IX.

Segment IX, in lateral view, long (Fig. 1A). Parapod narrow, simple, with small dorsolaterally directed tooth-like projections and short, stout setae (Figs 1A, 1B, 1C). Filipod long, slender, with elongate setae along length, apex attenuate (Fig. 1A). Preanal appendage short, rounded, setose (Figs 1A, 1C). Inferior appendage, first article long, slightly widened apically, otherwise relatively narrow and of uniform width, with subtriangular apical process, nearly equaling second article in length (Figs 1A, 1D); second article small, with subacute apex and small rounded carina ventrally (Figs 1A, 1D). Proctiger, in lateral view, broadly widened apically, with truncate apical margin, covered with minute setae, apicodorsal margin with long setae (Fig. 1A). Phallic apparatus complex (Fig. 1E, 1F); phallotheca broadly rounded basally, with narrow ventral process articulating with inferior appendages (Fig. 1E); posteriorly divided into long, paired processes (Fig. 1F); processes apically upturned and narrow, covered with minute setae and bearing spines on lateral edges (Figs 1E, 1F); aedeagus an elongate, stout, spine-like structure, with slight ventral curvature near base (Fig. 1E).

#### Holotype male.

**BRAZIL: Rio de Janeiro:** Cachoeiras de Macacu, Rio Souza, 16°26.567'S, 42°37.957'W, 150 m, 16.iii.1996, Holzenthal, Rochetti & Oliveira (pinned) (UMSP000031906) (MZSP).

#### Paratypes.

**BRAZIL: Rio de Janeiro:** same data as holotype, 23 females (pinned) (17 females, UMSP; 2 females, NMNH; 2 females, MZSP; 2 females, DZRJ).

#### Etymology.

This new species is named in honor of Dr. Roger Blahnik, who identified this new species.

### Atopsyche (Atopsyche) parauna sp. n.

urn:lsid:zoobank.org:act:D90947CD-CF4E-44EA-9819-D47E65CCF7F6

http://species-id.net/wiki/Atopsyche_parauna

Figs 2 , 5 , 8

#### Diagnosis.

This new species is most similar to *Atopsyche jaba* Blahnik and Gottschalk, described from Costa Rica. These two species share a similar mesal process on the first article of the inferior appendage and the complex phallotheca, with paired processes posteriorly. *Atopsyche parauna* sp. n. can be distinguished from *Atopsyche jaba* and other *Atopsyche* species by the broad parapod, in lateral view, with the dorsolateral margin serrate and with a midlateral spinose projection, whereas in *Atopsyche jaba*, the parapod has 2 prominent spines. In addition, the second article of the inferior appendage is shorter and slightly hooked in the new species, and the phallotheca is posteriorly divided into 2 long, paired processes, the dorsal one birfucate apically and the ventral one curved mesally.

#### Male.

Forewing length 5.0–5.5 mm (n=10). Overall body color brown; antennal scape brown, with long stramineous setae, pedicel brown, basal flagellomeres yellow, apical flagellomeres brown; setae of palps yellow; frons and vertex of head with long, erect brown and whitish setae; legs yellowish brown, coxae of all legs darker brown. Forewings brown; erect setae on veins forming distinct mottled pattern of alternate dark brown and yellow setae, with dark brown setae along costal margin; apex of wing with fringe of alternating patches of dark brown and yellow setae. Forewing venation complete (Fig. 5A); R1 branched; stem of fork I equal to its length; fork II long, sessile; stem of fork III equal to its length; fork IV long, sessile; stem of M slightly curved between m-cu crossvein and first fork of M; fork V long, narrow; Cu2 long, converging near fused anal veins, crossvein near apex forming small cell on posterior margin of wing; apex of fused anal veins very short. Hind wing (Fig. 5B) with Sc and R1 fused apically; forks I, III, and V present, the first with a short stem, the last long with a short stem, stem of fork III longer than its length; forks II and IV absent; M3+4 reaching wing margin; Cu2 long and almost straight; 1A long and strongly curved, with row of elongate setae along its length. Nygmas indistinct in both wings. Terga III and IV with pair of prominent rounded glands at anterolateral margin, lined internally with minute setae (Fig. 8A). Sternum V with pair of small rounded glands on anterolateral margins, with a keel-like projection (Fig. 8B). Sterna VI and VII with prominent spine-like ventral processes on posteromesal margins.

#### Male genitalia.

**Figure 2.**
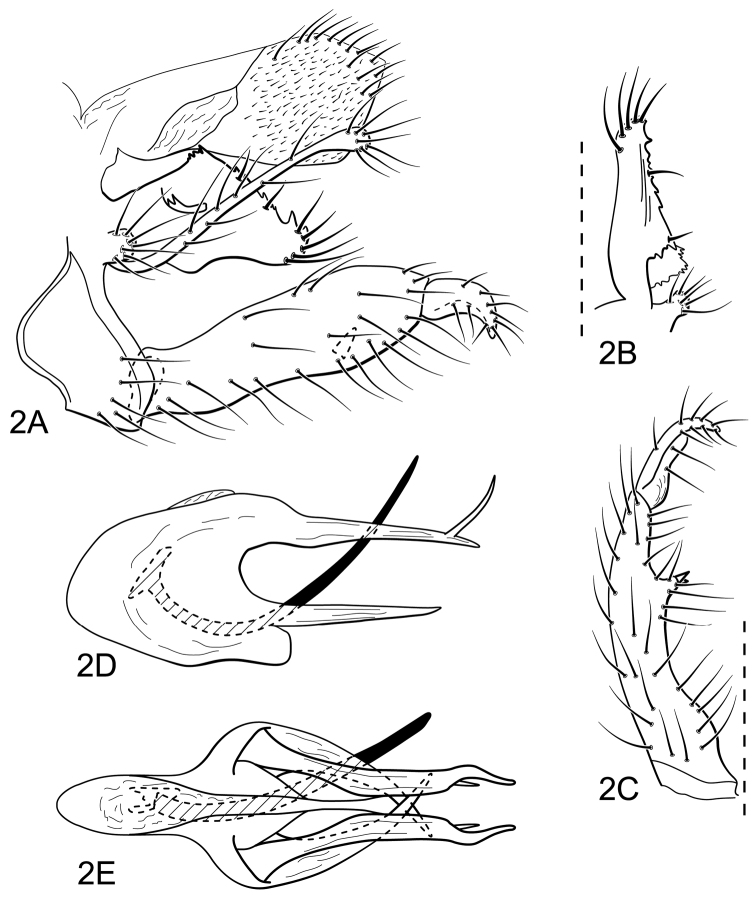
*Atopsyche (Atopsyche) parauna* sp. n. Male genitalia: **A** lateral **B** parapod and preanal appendage, dorsal **C** inferior appendage, ventral **D** phallic apparatus, lateral **E** phallic apparatus, dorsal.

Segment IX, in lateral view, short (Fig. 2A). Parapod broad, with dorsolateral margin serrate, setose, and with small, midlateral spinose projection (Figs 2A, 2B). Filipod long, slender, with elongate setae along length, apex somewhat capitate (Fig. 2A). Preanal appendage short, rounded, setose (Figs 2A, 2B). Inferior appendage, first article long, slightly constricted mesally, otherwise relatively narrow and of uniform width, without apical process, but mesally with small process at midlength, bearing short spines (Figs 2A, 2C); second article small, slightly hooked apically (Figs 2A, 2C). Proctiger, in lateral view, broadly widened apically, with angulate apical margin, covered with minute setae, apicodorsal margin with long setae (Fig. 2A). Phallic apparatus complex (Figs 2D, 2E); phallotheca broadly rounded basally, with short rounded ventral process articulating with inferior appendages (Fig. 2D); posteriorly divided into two long, paired processes, dorsal one longer and bifurcate apically, ventral one curved mesally (Figs 2D, 2E); aedeagus an elongate, stout, spine-like structure, with ventral curvature near base (Fig. 2D).

#### Holotype male.

**BRAZIL: Minas Gerais:** Rio Paraúna, 3 km S Santana do Riacho, 19°10.986'S, 43°43.485'W, 650 m, 11.xi.2001, Holzenthal, Paprocki, Blahnik & Amarante (pinned) (UMSP000080716) (MZSP).

#### Paratypes.

**BRAZIL: Minas Gerais:** same data as holotype, 2 males, 3 females (pinned) (UMSP); Cardeal Mota, Cachoeira Véu da Noiva, 19°18.912'S, 43°36.260'W, 800 m, 12.xi.2001, Holzenthal, Paprocki, Blahnik & Amarante, 2 males (pinned) (DZRJ); Rio Cipó, Cachoeira de Baixo, 19°20.553'S, 43°38.531'W, 750 m, 10.xi.2001, Holzenthal, Paprocki, Blahnik & Amarante, 3 males (pinned) (2 males, NMNH; 1 male, UMSP).

#### Etymology.

This species is named after the river where the holotype was collected, that means “black river” in the Tupi-guarani language.

### Atopsyche (Atopsaura) galharada sp. n.

urn:lsid:zoobank.org:act:4664284A-65D7-4F52-8ADB-8E66C1EAF4C2

http://species-id.net/wiki/Atopsyche_galharada

Figs 3 , 6 , 9

#### Diagnosis.

This is a distinctive new species in the genus, belonging to the *Atopsyche longipennis* Ulmer group of the subgenus *Atopsaura*. *Atopsyche galharada* sp. n. resembles *Atopsyche ayahuaca* Schmid and *Atopsyche plancki* Marlier in the absence of filipods and in the simple phallotheca. The new species can be distinguished from the others by the broader parapod with 2 carinas bearing tooth-like processes. The new species and *Atopsyche plancki* are similar in the short and broad first article of the inferior appendage, with an apical process, but in the new species this process is longer and the second article of the inferior appendage is broad in ventral view, and slightly hooked in lateral view. Also, *Atopsyche galharada* has the proctiger with a broad lateral sclerotized projection, and the phallotheca posteriorly divided into long, paired flangelike processes, broadest subapically, in dorsal view.

#### Male.

Forewing length 6.5–7.5 mm (n=23). Overall body color brown; antennal scape light brown, with long stramineous setae, pedicel brown, basal flagellomeres yellow, apical flagellomeres brown; setae of palps dark brown; frons and vertex of head with long, erect stramineous setae; legs yellowish brown, coxae of all legs darker brown. Forewings brown; erect setae on veins forming irregular pattern of alternate dark brown and yellow setae, with dark brown setae along costal margin; apex of wing with fringe of alternating patches of dark brown and yellow setae. Forewing venation complete (Fig. 6A); R1 branched; stem of fork I equal to its length; fork II long, sessile; stem of fork III equal in length to stem of fork I; fork IV long, sessile; stem of M slightly curved between m-cu crossvein and first fork of M; fork V long, narrow; Cu2 long, converging near fused anal veins, crossvein near apex forming small cell on posterior margin of wing; apex of fused anal veins very short. Hind wing (Fig. 6B) with Sc and R1 fused apically; forks I, III, and V present, the first with a short stem, the last long with a short stem, stem of fork III equal to its length; forks II and IV absent; M3+4 reaching wing margin; Cu2 long and almost straight; 1A long and sinuate. Nygmas indistinct in both wings. Sternum V with pair of small rounded glands on anterolateral margins, with a keel-like projection (Fig. 9). Sterna VI and VII with prominent spine-like ventral processes on posteromesal margins.

#### Male genitalia.

**Figure 3.**
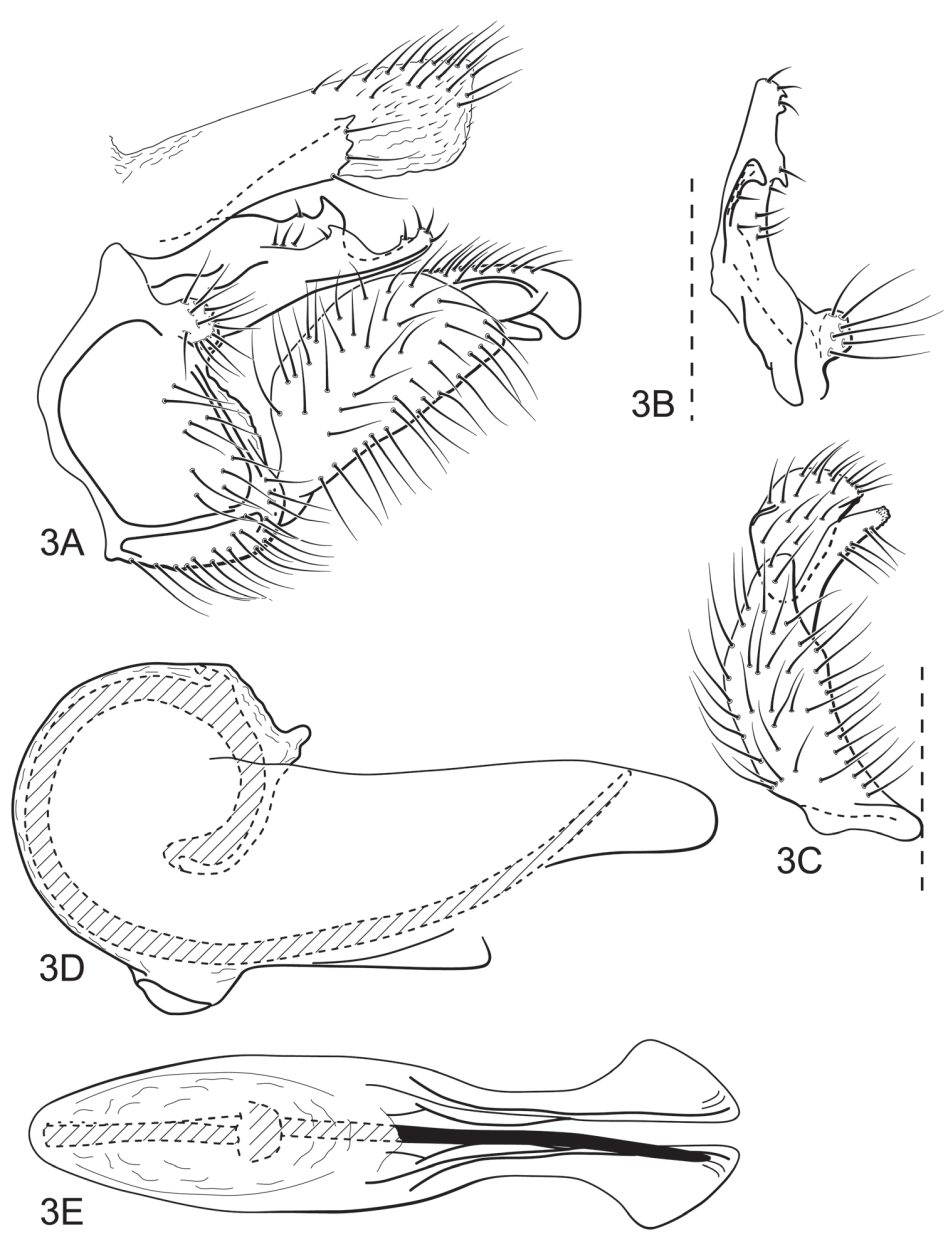
*Atopsyche (Atopsaura) galharada* sp. n. Male genitalia: **A** lateral **B** parapod and preanal appendage, dorsal **C** inferior appendage, ventral **D** phallic apparatus, lateral **E** phallic apparatus, dorsal.

Segment IX, in lateral view, long (Fig. 3A). Parapod broad basally, narrow apically, with two carinas bearing tooth-like processes along dorsolateral edges and short, stout setae (Figs 3A, 3B). Filipod absent (Fig. 3A). Preanal appendage short, rounded, setose (Figs 3A, 3B). Inferior appendage, first article short, uniformly broad, with narrow apical process, nearly equaling second article in length (Figs 3A, 3C); second article large, bulging basally and downturned apically, in ventral view, broad (Figs 3A, 3C). Proctiger, in lateral view, uniformly wide, with broad lateral sclerotized projection and truncate apical margin, covered with minute setae, apicodorsal margin with long setae (Fig. 3A). Phallic apparatus simple (Figs 3D, 3E); phallotheca broadly rounded basally, not articulating with inferior appendages (Fig. 3D); posteriorly divided into long, paired flangelike processes, broadest subapically, in dorsal view (Fig. 3E); aedeagus an elongate, stout, spine-like structure, with strong basal loop (Fig. 3D).

**Figures 4–6.**
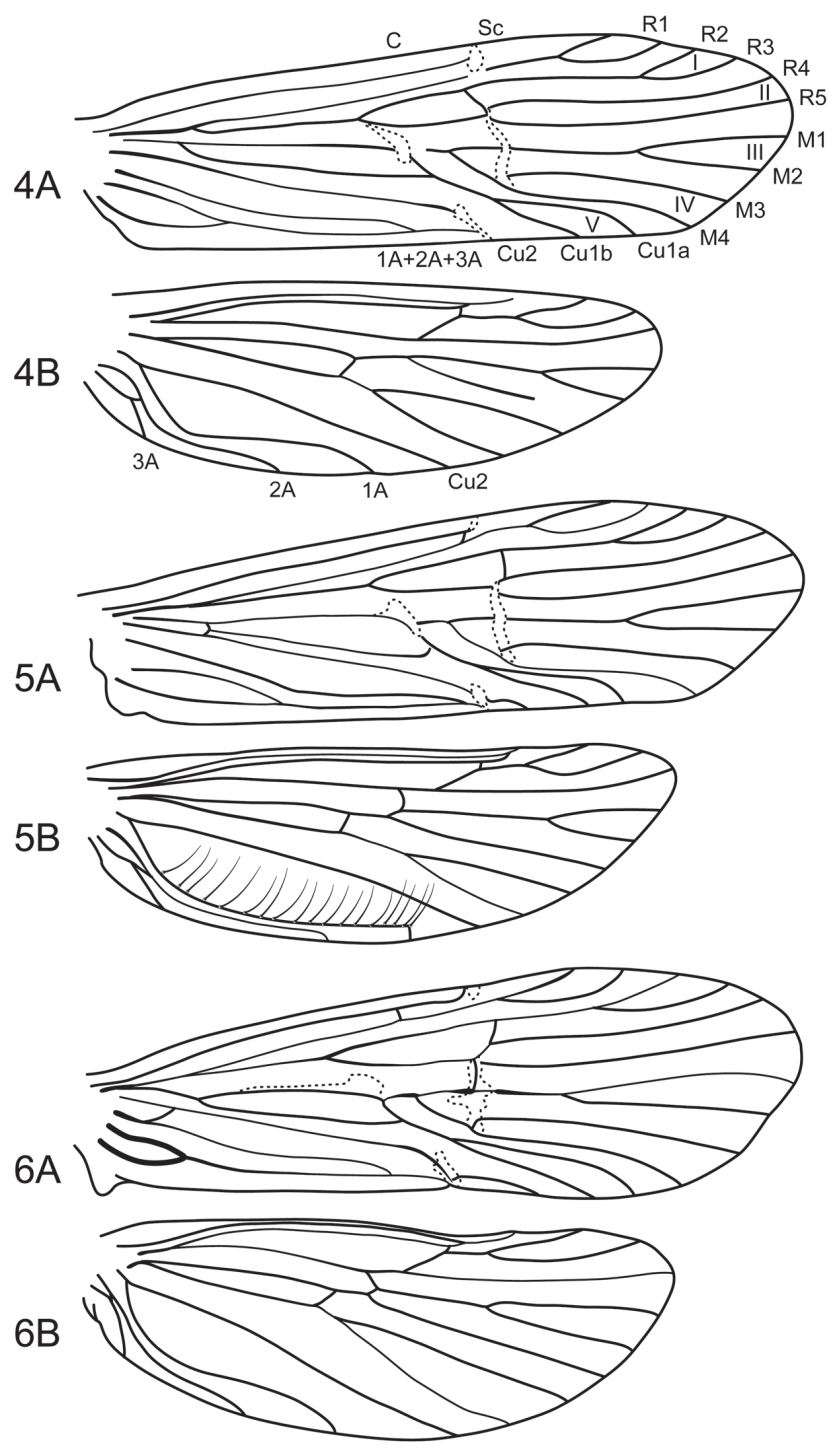
*Atopsyche* spp. n. Male wing venation: Figure **4** *Atopsyche (Atopsaura) blahniki* sp. n. **4A** forewing **4B** hind wing. Figure **5** *Atopsyche (Atopsyche) parauna* sp. n. **5A** forewing **5B** hind wing. Figure **6** *Atopsyche (Atopsaura) galharada* sp. n. **6A** forewing **6B** hind wing.

**Figures 7–9.**
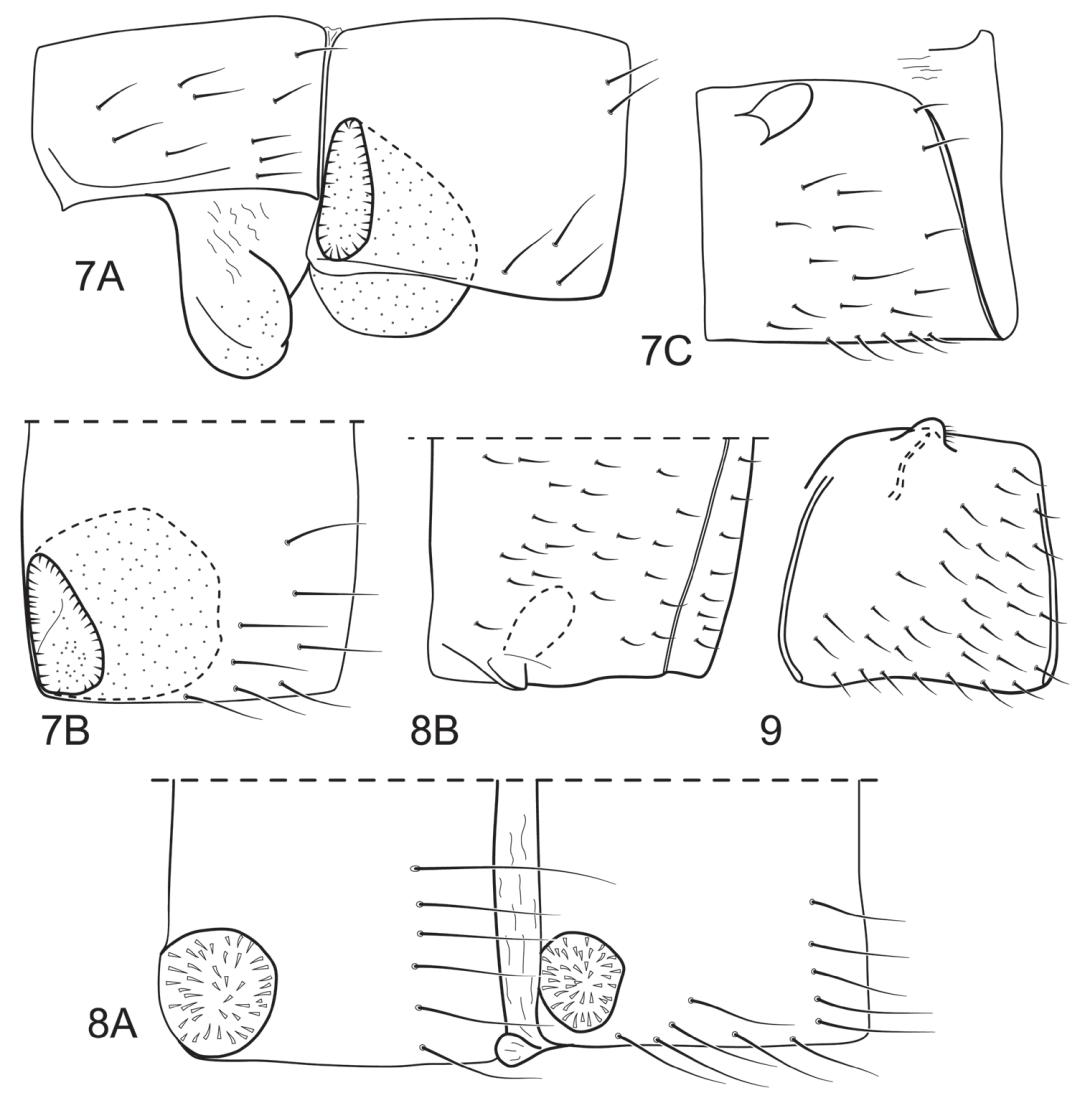
*Atopsyche* spp. n. Abdominal glands: Figure **7** *Atopsyche (Atopsaura) blahniki* sp. n. **7A** terga II and III, lateral **7B** tergum III, dorsal **C** sternum V, lateral. Figure **8** *Atopsyche (Atopsyche) parauna* sp. n. **8A** terga III and IV, dorsal **8B** sternum V, ventral. Figure **9** *Atopsyche (Atopsaura) galharada* sp. n., sternum V, lateral.

#### Holotype male.

**BRAZIL: São Paulo:** Campos do Jordão, Parque Estadual de Campos do Jordão, Rio Galharada, 22°41.662'S, 45°27.783'W, 1530 m, 4–5.iii.1996, Holzenthal & Guahyba (pinned) (UMSP000031880) (MZSP).

#### Paratypes.

**BRAZIL: São Paulo:** same data as holotype, 2 females (pinned) (MZSP); same data, except 22.i.1998, Holzenthal, Froehlich & Paprocki,17 females (pinned) (UMSP); same data, except 13–15.ix.2002, Blahnik, Prather, Melo, Huamantinco, 1 male, 3 females (alcohol) (UMSP); Parque Estadual de Campos do Jordão, Campo do Meio, 22°41.750'S, 45°29.448'W, 1500 m, 21.i.1998, Holzenthal, Froehlich & Paprocki, 2 females (pinned) (UMSP); same data, except 6.iii.1996, Holzenthal & Guahyba, 1 male, 2 females (pinned) (NMNH); Rio Casquilho, 3.4 km NE Parque Estadual de Campos do Jordão, 22°40.29'S, 45°27.87'W, 1550 m, 23.1.1998, Holzenthal, Froehlich & Paprocki, 18 males, 39 females (pinned) (15 males, 34 females, UMSP; 3 males, 5 females, DZRJ); Ribeirão do Casquilho, Bosque Vermelho, ca. 5 km Parque Estadual de Campos do Jordão, 22°40'S, 45°27.5'W, 1435 m, 16.ix.2002, Blahnik, Prather, Melo & Huamantinco, 3 males (UMSP).

#### Etymology.

This species is named after the river where holotype was collected.

### Atopsyche (Atopsyche) urumarca Schmid

http://species-id.net/wiki/Atopsyche_urumarca

Figs 10

Atopsyche (Atopsaura) urumarca Schmid, 1989: 131 [type locality: Brazil, Serra do Cipó, Rio Capivara; MZSP; male].

#### Diagnosis.

*Atopsyche urumarca* Schmid was described from Minas Gerais state, Brazil, and placed in the *Atopsyche bolivari* Banks group. According to Schimid (1989), *Atopsyche urumarca* is most similar to *Atopsyche pachacutec* Schmid, especially in the form of the parapod and the apex of the phallotheca, but they differ in the structure of the second article of the inferior appendage. We have examined material from southeastern Brazil, including the type locality, and we found an interesting populational variation in the male genitalia structure. This variation is noticeable in the shape of the parapod and the apex of phallotheca. In all specimens, the parapod, in lateral view, is broad basally, with an oblique, mesal U-shaped incision, but this incision is deepest in specimens collected in Ipoema, Minas Gerais state, and shallow in specimens from São Paulo state; the parapod also can end in a narrow and rounded apex or bear a small, dorsal spine. The phallotheca is broadly rounded basally, with 3 paired processes posteriorly, the dorsal one long and broad, with small dorsal and ventral projections at its apex. Some specimens have a small, additional spine-like process on the posterior margin of this dorsal process, and the ventral margin can be rounded, slightly pointed, or pointed and hooked apically.

**Figure 10.**
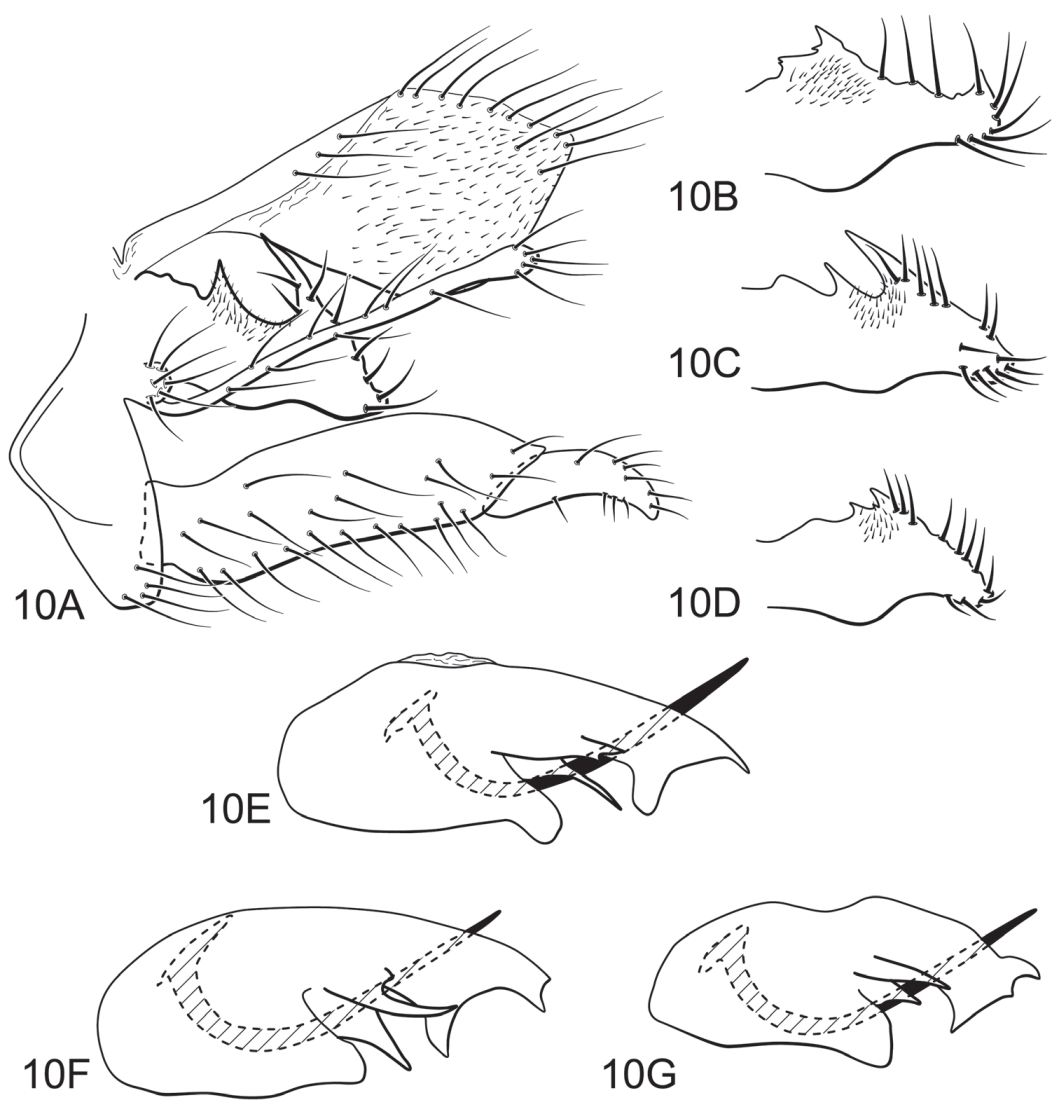
*Atopsyche (Atopsyche) urumarca* Schmd. Variation in male genitalia. **A** lateral, specimen from Ipoema, Minas Gerais **B–D** parapod, lateral **B** specimen from Serra do Cipó, Minas Gerais **C** specimen from Morro do Pilar, Minas Gerais **D** specimen from Altinópolis, São Paulo **E–G** phallic apparatus, lateral **E** specimen from Ipoema **F** specimen from Morro do Pilar **G** specimen from Altinópolis.

#### Material examined.

**BRAZIL: Minas Gerais:** Serra do Cipó, Rio Capivara, 19°20.553'S, 43°38.531'W, 950 m, 11.ii.1998, Holzenthal & Paprocki, 2 males (pinned) (UMSP); Serra do Cipó, trib. to Rio Capivara, 19°14.396'S, 43°34.939'W, 1000 m, 18.ii.1998, Holzenthal & Paprocki, 3 males (pinned) (MZSP); Rio Tanque, ca. 12 km (rd) from Ipoema, 19°32.208'S, 43°26.878'W, 750 m, 16.v.1998, Holzenthal & Paprocki, 2 males, 1 female (pinned) (DZRJ); Rio Santo Antônio, downstream from Morro do Pilar, 19°08.134'S, 43°21.256'W, 530 m, 17.x.2000, Paprocki & Ferreira, 1 male (pinned) (UMSP); P.E. de São Gonçalo do Rio Preto, Rio Preto, 18°07.841'S, 43°20.246'W, 791 m, 12.x.2000, Paprocki, Amarante, Salgado, 1 male (alcohol) (UMSP); **São Paulo:** Altinópolis, Cachoeira dos Macacos, 20°55.390'S, 47°22.758'W, 758 m, 18.xi.2003, Holzenthal, Paprocki & Calor, 27 males, 15 females (pinned) (UMSP), 9 males, 1 female (alcohol) (DZRJ); Altinópolis, Fazenda São João da Mata, Rio Baguassu, 21°00.588'S, 47°28.900'W, 745 m, 19–21.xi.2003, Holzenthal, Paprocki & Calor, 8 male, 17 females (pinned) (3 males, 5 females, NMNH; 5 males, 12 females, UMSP), 28 males (alcohol) (UMSP); Pedregulho, Sítio Bruninho, 20°09.240'S, 47°30.704'W, 630 m, 17.xi.2003, Holzenthal, Paprocki & Calor, 1 male, 1 female (pinned) (UMSP); Pedregulho, Ribeirão São Pedro, 20°09.113'S, 47°30.626'W, 617 m, 16.ix.2003, Holzenthal, Paprocki, Calor, 1 male (alcohol) (UMSP).

### Atopsyche (Atopsaura) plancki

Marlier

http://species-id.net/wiki/Atopsyche_plancki

Atopsyche (Atopsaura) plancki Marlier 1964: 2 [type locality: Brazil, São Paulo; IRSNB; male] - Blahnik et al. 2004: 4 [distribution; Brazil: Minas Gerais and Rio de Janeiro states]

#### Material examined.

**BRAZIL: Santa Catarina:** Parque Ecológico Spitzkopf, confl. Rio Ouro & Rio Caeté, 27°00.352'S, 49°06.693'W, 140 m, 25.xi.2003, Holzenthal, Paprocki, Calor, 1 male (alcohol) (UMSP).

## Supplementary Material

XML Treatment for Atopsyche (Atopsaura) blahniki

XML Treatment for Atopsyche (Atopsyche) parauna

XML Treatment for Atopsyche (Atopsaura) galharada

XML Treatment for Atopsyche (Atopsyche) urumarca

XML Treatment for Atopsyche (Atopsaura) plancki
